# Visceral leishmaniasis (Kala-azar) in Qom Province, Iran: Report of two cases

**DOI:** 10.12688/f1000research.15805.3

**Published:** 2019-08-19

**Authors:** Leyli Zanjirani Farahani, Abedin Saghafipour, Mehdi Mohebali, Behnaz Akhoundi, Hedayatollah Raufi

**Affiliations:** 1 Department of Medical Parasitology and Mycology, Tehran University of Medical Sciences, Tehran, Iran; 2 Department of Public Health, Qom University of Medical Sciences, Qom, Iran; 3 Health Center of Qom Province, Qom University of Medical Sciences, Qom, Iran

**Keywords:** Visceral leishmaniasis, Kala-azar, Qom

## Abstract

Visceral leishmaniasis (VL) is a fatal parasitic zoonotic worldwide disease, which transmits to humans by the infected *Phlebotomine* sand fly bite. The common form of VL in Iran is the Mediterranean type with the causative agent of *Leishmania infantum*, whose main reservoirs are stray and domesticated dogs. The disease has several endemic foci in Iran, mostly seen among children under the age of 10, living in rural areas and nomadic tribes. The first cases of Kala-Azar in Qom province, central Iran, were reported in the year 2001, from the villages of Ghahan district. After conducting VL control strategies in the area, no new cases of the disease had been reported until recently. The cases described here are two 2-year-old girls, living in the urban parts of Qom province, one of whom did not have a history of traveling to known endemic areas of the disease. The patients were admitted to hospital in 2016-2017, complaining from recurrent fever with unrecognized reason, associated with decreased appetite and weight loss. Disease follow-up demonstrated anemia and splenomegaly, which led to diagnosis of VL, and both patients are now fully recovered. VL was presumed to be controlled in Qom province but the present cases indicate that possible VL existence remains in the region. Therefore, urgent studies and periodic monitoring are needed to identify potential reservoirs of VL in the area.

## Introduction

 Human parasitic infections are important health issues due to insufficient epidemiological studies and lack of adequate information on the aspects of diagnosis and treatment. Problems caused by parasitic diseases in different regions have their own special characteristics. Visceral leishmaniasis (VL) or Kala-azar is one of the most severe zoonotic diseases caused by different species of *Leishmania,* which leads to death in 95% of cases if not treated ^1^. An estimated 50,000–90,000 new cases of VL occur worldwide annually ^2^. VL has been linked to hygiene and environmental health, along with malnutrition, weak immune system and population displacement considerations. VL is commonly observed in children in the under 10 year age group, especially 1–5 year-olds, but also afflicts adults suffering from immunodeficiency ^3^. The zoonotic form of VL is caused by *Leishmania infantum* whose pathogenic form transmits from the animal reservoir to humans through infected *Phlebotominae* sand fly bites. The parasite’s natural reservoirs are dogs, wild canids, foxes, jackals and occasionally wolves ^4^.

VL is seen most commonly in rural areas and clinical symptoms vary from asymptomatic forms and restricted infection to lethal VL. Disease incubation period lasts from a few weeks to several months ^5^. In Iran, fever and anemia have been reported as the most common clinical signs and hepatosplenomegaly is generally displayed six months after the onset of the infection. Bone marrow involvement causes severe anemia and cachexia in the patient. Finally, secondary bacterial infections can result in the patient’s death. VL clinical diagnosis is difficult due to nonspecific symptoms similar to other diseases, such as malaria, typhoid fever, brucellosis, lymphoma and leukemia, especially in non-endemic regions ^5, 6^.

Between 1998 and 2012 in Iran, 2632 cases of VL were recorded, with the majority of cases in the northern and southern parts of the country. The highest number of cases were in April and July in the age group 1–3 years and the annual average over the 14-year period was 175.4 cases. While the peak incidence was recorded in 2000 (13.15% of total leishmaniasis cases), VL occurrence decreased in the following years. The first cases of VL in Qom province were reported in 2001 and no new case has been reported until recently ^5^.

In this Clinical Practice Article, two cases of Kala-azar are reported, which were detected in Hazrat-E-Masoumeh Hospital in Qom Province, Iran, in 2016 and 2017.

## Case 1

In February 2016 a 22-month-old girl, who was living in Qom’s downtown, was admitted to Hazrate-E-Masoume Hospital with irregular prolonged fever, cough and loss of appetite for about one month. In the initial follow-up, the cause of fever remained unrecognized and the patient was referred to the hospital, accordingly. Based on her parent’s statement, the child had travelled to Dastgerd village, in Kahak district, south of Qom Province, in November 2016, two months before clinical signs appear.

**Figure 1.**
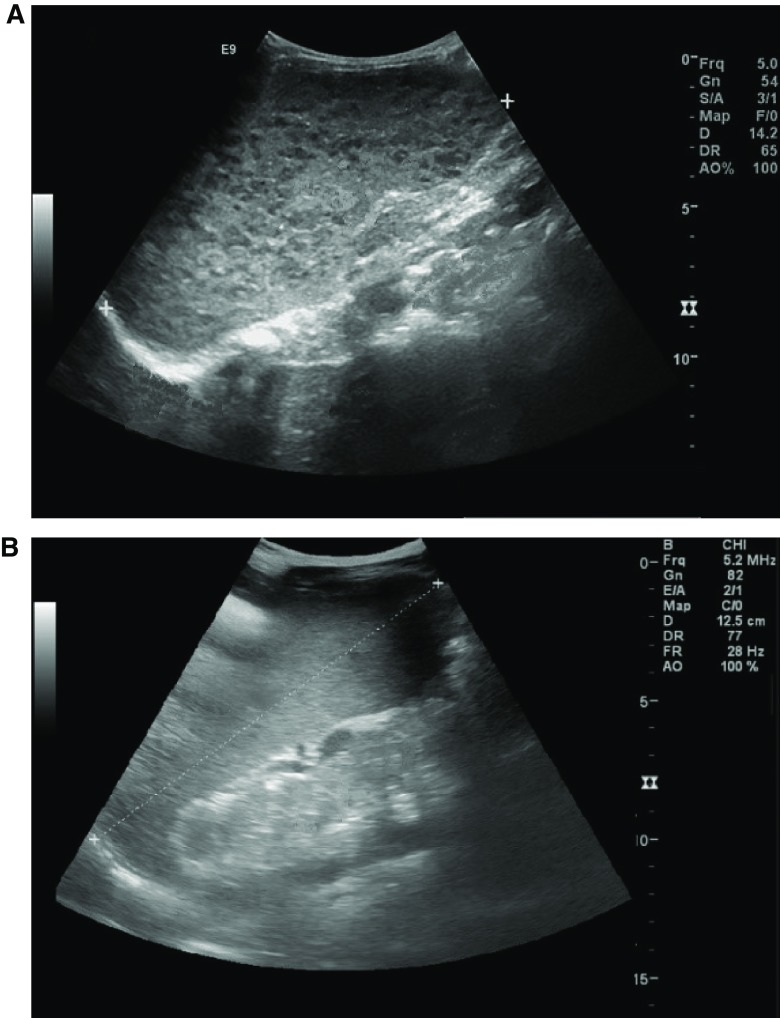
Ultrasound scan of enlarged spleen in ( **A**) Case 1 and ( **B**) Case 2.

In early clinical examinations, the patient’s throat, ears, heart and lungs were functioning normally. Abdominal ultrasonography showed normal liver tissue and enlarged spleen with diffused nodules and 14.5 mm spleen span ( Figure 1). Blood smear examination showed hypochromic microcytic anemia with white blood cell and platelet number reduction ( Table 1).

**Table 1.** Laboratory blood test results of the cases on admittance and one month after treatment.

	Case 1		Case 2		
	Before diagnosis	One month after treatment	Before diagnosis	one month after treatment	Normal range
Hemoglobin (g/dl)	10.5	11.2	8.9	12	11–14
Hematocrit (%)	28	31	25	33	31–41
Leukocytes (/mm ^3^)	3400	6600	2900	6200	6000–17000
Neutrophil (%)	35	34	30	35	35–80
Lymphocyte (%)	62	56	66	60	35–80
Platelet count (/mm ^3^)	118000	252000	83000	285000	150000–400000
ESR (mm/hour)	82	50	110	62	0–10
CRP	1+	Negative	2+	Negative	Negative

Serological tests for human immunodeficiency virus (HIV), hepatitis and malaria showed negative results. Blood culture, tuberculin test and thoracic radiography showed no specific cause for the fever. Due to associated fever with enlargement of the spleen and pancytopenia, Direct Agglutination Test (DAT) was performed to detect anti-Leishmania antibodies, which showed positive result with a high titer of anti- *L. infantum* antibodies as 1:6400 which was confirmed with Indirect Immunofluorescence Assay (IFA>1:640).

After diagnosis, Amphotericin-B injection was prescribed at 1 mg/kg for the first day, increased to 5 mg/kg during one week. The last dose was continued until day 10. As soon as treatment began, the patient’s fever reduced and the patient’s general state improved. In the next follow-up, two weeks later, the blood cell count had risen and the patient was considered successfully treated.

## Case 2

In April 2017 a 26-month-old girl was admitted to Hazrat-E-Masoumeh Hospital in Qom. The patient lived in Qom city, and had no history of travelling to VL endemic regions since she was born. The patient presented with an unknown, persistent fever, anorexia, and general weakness, which had started four months ago, not responding to antibiotic therapy. The patient had some bruises on her abdomen and legs that appeared a month earlier, which caused the physicians to suspect anemia and leukemia.

**Figure 2.**
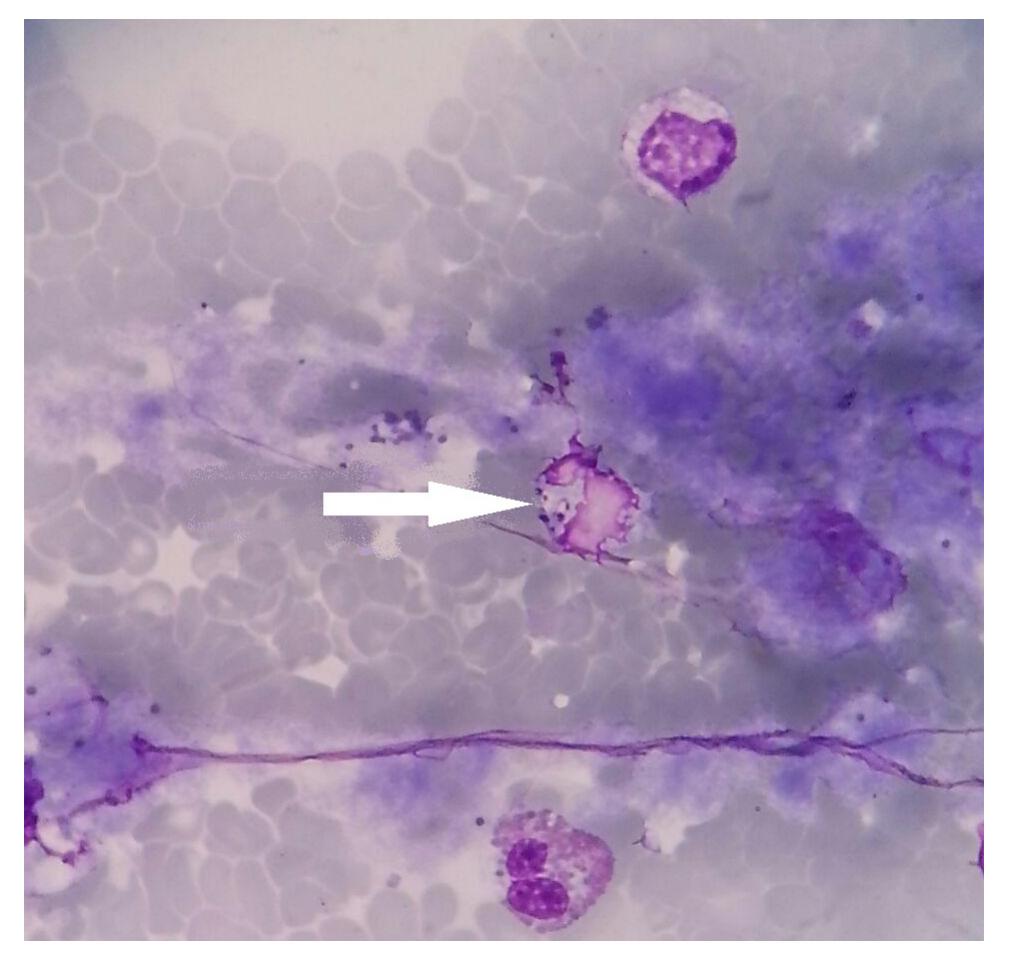
Bone marrow aspiration of case 2, showing Leishmania amastigots within macrophage.

Ultrasonography demonstrated mild enlargement of the spleen ( Figure 1). Examinations showed reduction in all blood elements ( Table 1). The results of typical serological tests were negative. Bone marrow aspiration was evaluated because of pancytopenia in which no blast cell was seen. Then, due to observation of amastigotes of *Leishmania* parasite (Leishman-Donovan bodies) within bone marrow macrophages, and the positive DAT result (>1:3200), visceral leishmaniasis was diagnosed ( Figure 2). Therefore, Amphotericin-B treatment was initiated with dose of 1 mg/kg for 21 days. After four days, the patient’s fever disappeared, her general condition improved and blood cell number increased at the next month follow-up.

## Discussion

Visceral leishmaniasis (VL) is endemic in some provinces of Iran, including Ardebil, East Azarbaijan, Bushehr, Fars, and North Khorasan, and sporadically occurs in other provinces. The first human VL case in Iran was observed in Northern Iran in 1949 ^3^. In Qom province, the first VL cases were observed in 2001, during the study by Fakhar *et al.*, in which 1.7% of 416 serum samples were diagnosed seropositive, and was related to 25% contamination of dogs in rural districts of Ghahan, a northwestern part of the province ^7^.

This article reports the first cases of Kala-azar in urban areas of Qom province. So far, over 95% of the cases in Iran have been from rural areas ^3^. Despite the history of traveling to a village in the first case presented here, the disease may not be linked to the village definitely. The probability of there being more patients in Qom province and referring them to hospitals out of the province requires further investigation.

Kala-azar is frequently misdiagnosed because of nonspecific symptoms. Also false negative results of serologic and microscopic examinations in the disease early stages, make the diagnosis an important issue, in non-endemic areas. Due to the lack of knowledge about VL, the cases were referred to hospital a few months after the onset of symptoms. Therefore, there is not enough available history of children like the presence of malnutrition or other underlying illness which may affect the immune system of cases.

The symptoms of VL are related to involvement of the reticuloendothelial system, which includes enlargement of the spleen, liver and lymph nodes. Moreover, bone marrow infection leads to a decrease in its normal activity, resulting in anemia, leukopenia, and thrombocytopenia ^6^. The common symptoms in the present patients were intermittent fever, pancytopenia and splenomegaly. In previous studies, the main hallmarks of the disease were persistent fever, pallor, and spleen and liver enlargement ^3^. Spleen enlargement is also seen in other infections like CMV, toxoplasmosis, mononucleosis, tuberculosis, malaria and hematological disorders. In the present cases no hepatomegaly was observed. Normochromic normocytic anemia and pancytopenia (decrease of all blood cell types) were features of identified anemia in patients in this report. The incubation period in VL varies from two weeks to several months. In one quarter of VL cases, VL develops actively and symptoms appear between 2–8 months after parasite entrance to the body ^6, 8^. There is no obvious evidence of exposure to the parasite in the cases presented here, although in the first case it can be attributed to the child’s visit to the villages of Qom. The role of domestic or stray dogs and *Phlebotominae* sand flies in transmission cycle of the disease is clear ^4^. None of the patient families had dogs but more research is needed to check the stray dogs infection. 

DAT and IFA serology methods are considered the most efficient diagnostic tests for Kala-azar. The definitive infection diagnosis is done through parasitological methods involving microscopic examination of spleen, bone marrow and lymph nodes aspiration to observe the parasite, which are sensitive but invasive and potentially hazardous ^9^. Evaluation of indirect IFA and DAT showed that IFA is more reliable in VL diagnosis than DAT but needs advanced laboratory equipment, while DAT is a simple, precise, cost effective and applicable test for all situations ^10^. Due to the possibility of cross-reaction between VL and cutaneous leishmaniasis, tuberculosis and malaria in performing DAT, the history of such diseases in the patient should be investigated as well as any blood transfusion history or congenital transmission. Upon referral of the first patient reported here, both DAT and IFA results were positive. Since false positive results are likely in DAT method, it is advised to be confirmed by using a definitive parasitology method such as bone marrow aspiration, which was performed in the second patient.

In a VL reservoir study in Iran, not only stray dogs, but also domestic dogs have been infected with *Leishmania* parasite. In 2012–2014, asymptomatic and symptomatic domestic dogs were compared in Meshkin-shahr; 18.6% of asymptomatic domestic dogs had VL infection, and surprisingly, 13.4% of the asymptomatic dogs demonstrated negative serology tests while they had been positive in the parasitology exam. Therefore, the presence of domesticated and stray dogs in urban and rural areas play an important role in the occurrence of sporadic cases of VL ^11, 12^. Because of the complex nature of VL manifestations and the risk of misdiagnosis, physicians in urban and rural health centers should remain vigilant, as they play a crucial role in early diagnosis and timely treatment of patients ^13^.

In Qom Province, central Iran, Kala-azar had presumably been controlled after control strategies were implemented, and no new cases had been reported recently. The two cases in this report indicate that VL might not be eradicated totally in this area. Therefore, further studies on epidemiological aspects of *Leishmania* reservoirs and vectors are recommended along with increased surveillance system awareness.

## Consent

Written informed consent for the publication of clinical details of the patients described here was obtained from their parents.

## Data availability

All data underlying the results are available as part of the article and no additional source data are required.
