# Supplementary material for: Visceral leishmaniasis (Kala-azar) in Qom Province, Iran: Report of two cases
Source: F1000Res. 2019 Aug 19;7:1371. Originally published 2018 Aug 31. [Version 3] doi: 10.12688/f1000research.15805.3 (PMC6593324; doi:10.12688/f1000research.15805.3)
Supplement: Peer review history

# Peer review: Reviewer response for version 2

**DOI:** 10.5256/f1000research.21218.r48933

**Published:** 2019-06-24

**Authors:** Aref Salehzadeh

**Affiliations:** 1 Department of Medical Entomology and Vector Control, School of Medicine, Hamadan University of Medical Sciences, Hamadan, Iran

The quality of the present version of the manuscript is good but there are some minor typographical errors. For instance:

- Please check and correct the sentence “ The role of domestic or stray dogs and Phlebotominae sand flies in the transmission cycle of the disease in clear”  in line 21-22 of the fourth paragraph of the discussion section.
- Please check and correct the word  “congenital” in line 14 of the 5 ^th^ paragraph of the discussion section

Please clarify which types of Amphotericin-B has been used (liposomal or …)

 Also, in line 4 of the 3 ^rd^ paragraph of discussion please add one or two related references.

I confirm that I have read this submission and believe that I have an appropriate level of expertise to confirm that it is of an acceptable scientific standard.

# Peer review: Reviewer response for version 2

**DOI:** 10.5256/f1000research.21218.r48934

**Published:** 2019-06-06

**Authors:** Joziana Muniz de Paiva Barçante, Thales Barçante

**Affiliations:** 1 Department of Health Sciences, Federal University of Lavras (UFLA), Lavras, Brazil

We confirm that we have read this submission and believe that we have an appropriate level of expertise to confirm that it is of an acceptable scientific standard.

# Peer review: Reviewer response for version 1

**DOI:** 10.5256/f1000research.17252.r45651

**Published:** 2019-04-25

**Authors:** Joziana Muniz de Paiva Barçante

**Affiliations:** 1 Department of Health Sciences, Federal University of Lavras (UFLA), Lavras, Brazil

Leishmaniasis is considered an important zoonotic disease worldwide. So, the publication is relevant. Some aspects might be considered before the indexing of the manuscript: 

1. Is necessary a revision regarding correct biological concepts and terms. The first phrase of introduction is not correct. Third paragraph: 13.15% of total leismaniasis instead of Leishmania.
2. Is necessary a better description of the Figure 1. Also, in the legend, indicating the enlargement of the spleen. 
3. Is it necessary to include a image of amastigota identified in case 2. 
4. The authors should better explore the conclusion.

I confirm that I have read this submission and believe that I have an appropriate level of expertise to confirm that it is of an acceptable scientific standard, however I have significant reservations, as outlined above.

# Peer review: Reviewer response for version 1

**DOI:** 10.5256/f1000research.17252.r37818

**Published:** 2018-09-05

**Authors:** Aref Salehzadeh

**Affiliations:** 1 Department of Medical Entomology and Vector Control, School of Medicine, Hamadan University of Medical Sciences, Hamadan, Iran

The table is not acceptable in present form and should be corrected accurately by an expert physician, for example, the platelet  number should be shown per ml (mm3), the leukocytes count should be present as number per ml, or hemoglobin level by g/dl.

 The authors should include a figure of bone marrow smear. The bone marrow aspirate is the gold standard for confirming diagnosis. A definite diagnosis requires the demonstration of the parasites in tissue smears of the affected organs.

 The details of patients are very brief and has been not described precisely for instance  in second paragraph of discussion authors have argued that “the cases were referred to hospital more than two months after the onset of symptoms” whereas such information is not seen in description of case one in page 3.

 Did the authors check the possibility of cutaneous leishmaniasis, tuberculosis and malaria infections in patients?

 What about **congenital** transmission?  Did the authors check mothers?  Because there are evidences that vertical transmission  might occur either transplacentally during pregnancy (in utero) or, most likely, during labor via blood exchange from the mother to the child (although in the vast majority of congenitally infected children, the symptoms and signs of VL develops during the first 12 months of life)

 Discussion is weak and should be rewritten and I cannot approve the manuscript in this form.

I confirm that I have read this submission and believe that I have an appropriate level of expertise to confirm that it is of an acceptable scientific standard, however I have significant reservations, as outlined above.

# Response

**Published:** 2019-01-26

**Authors:** Leyli Zanjirani Farahani

**Affiliations:** Tehran University of Medical Sciences, Iran

Referee response:

 1-The table is not acceptable in present form and should be corrected accurately by an expert physician, for example, the platelet number should be shown per ml (mm3), the leukocytes count should be present as number per ml, or hemoglobin level by g/dl.

 Many thanks for your comment, we will correct the table as you mentioned.

 2-The authors should include a figure of bone marrow smear. The bone marrow aspirate is the gold standard for confirming diagnosis. A definite diagnosis requires the demonstration of the parasites in tissue smears of the affected organs.

 Thanks for your comment, we agree you in this matter. But because of the onset of this manuscript after the patient recovery and they were discharged from the hospital, no figure of bone marrow slide was obtained while diagnosing leishmaniasis. Since the blood smears are saved in the laboratory archive, we can photograph them and add it to the article with the editor approve.

 3-The details of patients are very brief and has been not described precisely for instance  in second paragraph of discussion authors have argued that “the cases were referred to hospital more than two months after the onset of symptoms” whereas such information is not seen in description of case one in page 3.

 Regarding your comment, the parents of patients had visited various physicians at the onset of the disease symptoms and unfortunately, they had no complete report of these visits. For this reason, the data before being admitted to the Hospital is not complete.

 4-Did the authors check the possibility of cutaneous leishmaniasis, tuberculosis and malaria infections in patients?

 Many thanks for your precision, we have discussed this issue in paragraph 4 of the discussion. Because of the possible interaction between some diseases such as malaria, tuberculosis and cutaneous leishmaniasis and VL diagnosis via DAT, the physicians had assessed it in primary examinations. Also there was no history of Kala-azar in the patients family members.

 5-What about **congenital** transmission?  Did the authors check mothers?  Because there are evidences that vertical transmission might occur either trans placental during pregnancy (in utero) or, most likely, during labor via blood exchange from the mother to the child (although in the vast majority of congenitally infected children, the symptoms and signs of VL develops during the first 12 months of life)

 As you mentioned, it would be important in the first 12 months of the life. But patients described here are older than two years. However, we would like to inform you that there was no history of Kala-azar in the family members of children.

 Respecting your comment, it should be noted that the antigen used in DAT method is prepared using *Leishmania infantum,* which is the agent of the Mediterranean Leishmaniasis and causes the disease in children. The probability of Mediterranean Leishmaniasis in an adult and congenital transmission with this high antibody titer at 2 years of age seems unlikely.

 6-Discussion is weak and should be rewritten and I cannot approve the manuscript in this form.

 Thanks for your valuable guidance. We would appreciate if you could help us with the shortcomings in the article's discussion. Since this manuscript is important due to reporting VL cases in urban areas of Qom province, central Iran, which had not been reported for long, we will do our best to complete the discussion and convince you.

# Response

**Published:** 2019-07-03

**Authors:** Leyli Zanjirani Farahani

**Affiliations:** Tehran University of Medical Sciences, Iran

Dear Dr. Aref Salehzadeh,

 Many thanks for your precious comments on our article. Here are our respond to your latest comments:

 1-Please check and correct the sentence “The role of domestic or stray dogs and Phlebotominae sand flies in the transmission cycle of the disease in clear” in line 21-22 of the fourth paragraph of the discussion section.

 The mistake is corrected.

 2-Please check and correct the word “congenital” in line 14 of the 5th paragraph of the discussion section.

 We checked the word “congenital” in the dictionary and on the internet but it seems correct, respecting your opinion.

 3-Please clarify which types of Amphotericin-B has been used (liposomal or …).

 Since the liposomal form of Amphotericin-B is known as “AmBisome®” we didn’t explain about the type of used Amphotericin-B in treatment of the patients.

 4-In line 4 of the 3rd paragraph of discussion please add one or two related references.

 Respecting your comment, there is only a report of both patients history in the mentioned lines which does not need any reference.
